# A Kalman Filter-Based Kernelized Correlation Filter Algorithm for Pose Measurement of a Micro-Robot

**DOI:** 10.3390/mi12070774

**Published:** 2021-06-30

**Authors:** Heng Zhang, Hongwu Zhan, Libin Zhang, Fang Xu, Xinbin Ding

**Affiliations:** Mechanical Engineering College, Zhejiang University of Technology, Hangzhou 310023, China; hengz067@zjut.edu.cn (H.Z.); lbz@zjut.edu.cn (L.Z.); fangx@zjut.edu.cn (F.X.); 15658092668@163.com (X.D.)

**Keywords:** Kalman filter, kernelized correlation filter, target tracking, machine vision, pose measurement

## Abstract

This paper proposes a moving-target tracking algorithm that measures the pose of a micro-robot with high precision and high speed using the Kalman filter-based kernelized correlation filter (K2CF) algorithm. The adaptive Kalman filter can predict the state of linearly and nonlinearly fast-moving targets. The kernelized correlation filter algorithm then accurately detects the positions of the moving targets and uses the detection results to modify the moving states of the targets. This paper verifies the performance of the algorithm on a monocular vision measurement platform and using a pose measurement method. The K2CF algorithm was embedded in the micro-robot’s attitude measurement system, and the tracking performances of three different trackers were compared under different motion conditions. Our tracker improved the positioning accuracy and maintained real-time operation. In a comparison study of K2CF and many other algorithms on Object Tracking Benchmark-50 and Object Tracking Benchmark-100 video sequences, the K2CF algorithm achieved the highest accuracy. In the 400 mm × 300 mm field of view, when the target radius is about 3 mm and the inter-frame acceleration displacement does not exceed 5.6 mm, the root-mean-square error of position and attitude angle can satisfy the precision requirements of the system.

## 1. Introduction

Technological developments in information, electronics and mechatronics have advanced the use of micro-robots in precision operation fields, such as complex assembly [1,2], advanced machining [3,4], intelligent manufacturing [5], automatic monitoring [6,7], nondestructive testing [8,9] and digital printing [10,11]. To accomplish a wide range of accurate operations, micro-robots such as miniature assembly robots and micro-robots for large-format digital printing must plan their motion trajectories and determine if there is a deviation in the path that requires accurate pose measurements in real-time [12,13,14]. It is important to measure the pose change of these robots for their trajectory planning and control effect detection. Pose measurement technologies of micro-robots are broadly divisible into relative localization and absolute localization [15,16,17]. Kelly and Martinoli located the relative position of a robot among a crowd of micro-mobile machines using an infrared transmitter and an infrared receiver. The positioning accuracy of their system was 40 cm within a measurement range of 3 m, and the maximum error in their attitude angle was 17.4° [18,19]. However, estimating the range from the amplitude of an incoming infrared signal is intrinsically limited to low-precision measurements. Qazizada designed an inertial navigation system with a gyroscope and an accelerometer. Although this system determines a robot’s current pose, it is unsuitable for long-term high-precision positioning [20]. Absolute localization usually requires beacons and global vision positioning. Diederichs detected, classified, located and tracked micro-mobile robots by a micro-camera, which achieves nanometer-level manipulation but requires the size and speed of the target [21]. Equipped with a target tracking algorithm, vision measurement is widely used for tracking and locating micro-robots with high precision and high speed under no-contact conditions [22,23].

With respect to the moving target tracking algorithms, they are generally divided into production model methods and discriminative model methods. The production model methods conduct modeling for the target region in the current frame and detect the most similar region in the next frame [24,25,26]. The classical algorithms mainly contain the particle filter tracking algorithm [27], mean shift algorithm [28] and optical flow algorithm based on feature point [29,30]. Production model tracking algorithms detect the target region by dense or sparse search of each frame image. Nevertheless, they are not suitable for fast detecting in large pixel images due to the high computation load. For the discriminative model methods, the target regions are used as positive samples and the background areas are regarded as negative samples, which are used to train the classifier [31,32,33]. Kalal proposed a tracking-learning-detection (TLD) algorithm for long time tracking, and performed adaptive processing for various scales, attitudes and illumination changes by offline learning [34]. For the tracking speed, detection accuracy and tracking success rate, the TLD algorithm is lower than the correlation filter tracking algorithm. Due to the high computational efficiency and cyclic dense sampling, the correlation filter tracking algorithm can realize high precision and high-speed tracking. Bolme developed an adaptive correlation filter to achieve visual object tracking, which could reach hundreds of frames per second [35]. Henriques presented a circulant structure of tracking-by-detection with kernels [36]. It adopted a cyclic matrix to achieve a dense sample and transformed computation into the frequency domain by Fourier transform. Then, using the histogram of oriented gradient (HOG) as a sample feature [37], the kernelized correlation filter (KCF) tracking algorithm is proposed [38]. Lim constructed a series of image sequence data with real geographical location and range to analyze the performance of various tracking algorithms and obtained the performance of each algorithm under the same evaluation criteria [39]. According to their results, the KCF tracking algorithm has no requirement for tracking objects. It can update the classifier and recognize the target by self-learning. Within a certain error threshold and overlapping threshold, it can perform fast detection for moving targets.

However, the KCF is very sensitive to occlusion, scale changes and fast motions. To overcome these difficulties, Li replaced the fixed-size template in the KCF tracker with an effective scale-adaptive KCF tracker [40]. In an experimental evaluation, the scale-adaptive tracker showed a promising ability to change the scale of a target. By integrating a Kalman filer with KCF, Huynh developed a new tracking method that overcomes occlusion and human-crossing [41]. The KCF in their method estimates the target position based on the Kalman filter prediction and updates the kernel model accordingly. When the tracker encounters an occlusion, the Kalman filter omits the observed values of the KCF and updates the state based on the previous state. This tracker properly handles occlusion and human-crossing tasks, but its Kalman filter cannot effectively predict the target information of fast-moving objects. When the target moves near or over the boundary, the object information will be filtered out.

Apparently, the boundary effect is the main problem in KCF tracking algorithms, as it risks error fluctuations and tracking loss, especially for fast-moving targets. To correct the boundary effect in the KCF tracking algorithm, this paper proposes an adaptive kernelized correlation filter (K^2^CF) algorithm that integrates the adaptive Kalman filter and can effectively predict the position of the target in different moving states. The predicted position is then refined to a precise precision by the KCF algorithm, and the moving state of the target is corrected based on the detection results. The K^2^CF algorithm reduces the kernel response value of the KCF algorithm and improves its detection accuracy and tracking stability. The least-square method and morphology processing method are used to extract the feature points of the tracking area, which further improves the tracking accuracy. The image coordinates and motion coordinates are transformed by the camera calibration principle to complete the pose measurement of the target. The method can measure the pose of the tiny moving target quickly and effectively, and the detection accuracy is high.

## 2. Pose Measurement Principle for a Micro-Mobile Robot

**Figure 1:**
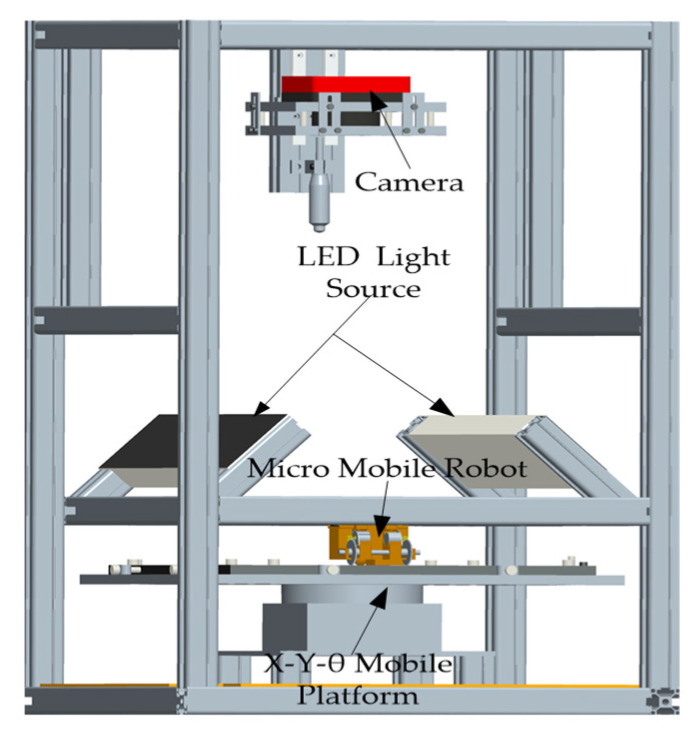
Visual detection platform for micro-mobile robot.

The pose of a micro-mobile robot in two-dimensional space is measured from the position information and direction angle in a moving plane. To meet the detection accuracy requirements of a mobile robot, we first design a high-precision visual detection platform (see Figure 1). Applying the principle of camera calibration, we then establish the mapping relationship between the moving plane and the image plane. Finally, we extract the feature points of the micro-robot in the field of view and acquire the position and direction angle of the moving target.

### 2.1. High-Precision Vision Measurement Platform

When a target moves at speeds below 0.4 m/s in a 400 mm × 300 mm range, the trajectory plan of a micro-mobile robot must resolve the motion to within 0.1 mm. That is, the precision of the vision detection platform must be at least 0.1 mm, and the moving speed of the target cannot exceed 0.4 m/s.

The camera in our system is an IO-Flare series 12M125C with a CMOS global shutter (Figure 1). The pixel size of the images is 4096 × 3072, and the frame rate is 124 fps. The acquisition card (AS-FBD-2XCLD-2PE8) meets the requisite high-rate acquisition through a camera-link serial bus. The uniform light source of the LED strip lamp is flanked on both sides. The required illumination is obtained by an external trigger control that ensures a target displacement not exceeding one pixel while imaging [42,43,44]. An *X–Y–θ* triaxial mobile platform, which simulates random movements in the two-dimensional plane, is used for verifying the accuracy of the detection algorithm and adjusting the field of view.

### 2.2. Visual Platform Calibration

**Figure 2:**
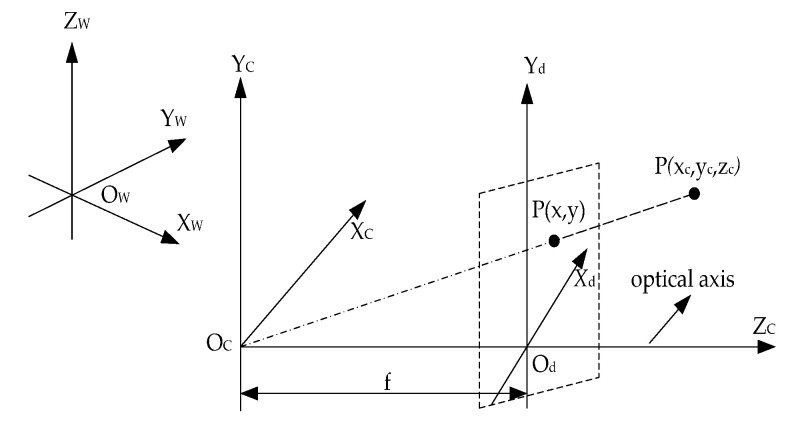
Camera imaging model for visual platform calibration.

The visual detection system is calibrated using various external and internal parameters. The internal parameters are the intrinsic parameters of the camera, which provide the conversion relationship between the camera and image coordinate systems [45,46,47]. The external parameters provide the relative position and attitude relationships between the camera and motion coordinate systems. The camera imaging model of the visual platform calibration is shown in Figure 2.

The image coordinates are converted to world coordinates as follows:

(1) $$Z_{c}\left(\begin{array}{l} u\\ v\\ 1 \end{array}\right)\;=\;\left(\begin{array}{l} \frac{f}{\mathrm{dx}}\;0\;u_{0}\;0\\ \;0\;\frac{f}{\mathrm{dy}}\;v_{0}\;0\\ \;0\;0\;1\;0 \end{array}\right)\left(\begin{array}{l} R_{1}\;T\\ 0\;1 \end{array}\right)\left(\begin{array}{l} X_{w}\\ Y_{w}\\ Z_{w}\\ \;1 \end{array}\right)$$

where (u,v) are the image coordinate, $\left(X_{W},Y_{W},Z_{W}\right)$ are the word coordinate, f is the focal length of the camera, dx and dy are the pixel sizes in the x and y directions, respectively, and $\left(u_{0},v_{0}\right)$ are the optical-center camera coordinates, $R_{1}$ and T are the rotation and moving matrices, respectively, $Z_{c}$ is the distance between the camera lens and the target, $M_{1}$ and $M_{2}$ are matrices of the internal and external parameters, respectively:

(2) $$M_{1}=\left(\begin{array}{l} \frac{f}{\mathrm{dx}}\;0\;u_{0}\;0\\ 0\;\frac{f}{\mathrm{dy}}\;v_{0}\;0\\ 0\;0\;1\;0 \end{array}\right)M_{2}=\left(\begin{array}{l} R_{1}\;T\\ 0\;1 \end{array}\right)$$

The camera calibration is mainly required for solving the perspective projections of $M_{1}$ and $M_{2}$ between the imaging and target planes. Therefore, if $M_{1}$ and $M_{2}$ are obtained, the world coordinates can be determined from the known image coordinates. For a mobile micro robot in a two-dimensional plane, we construct an $X_{C}O_{C}Y_{C}$ coordinate system with origin placed at the starting point of the mobile robot.

**Figure 3:**
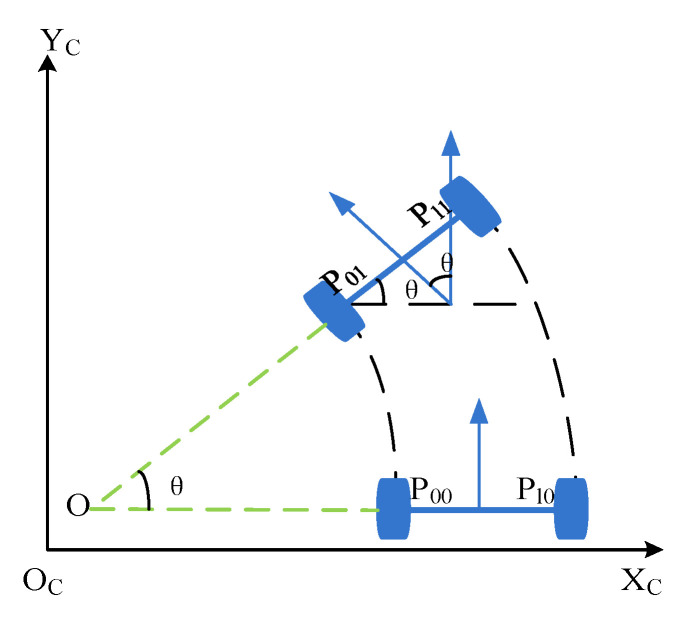
Target pose in a 2-dimension plane.

As shown in Figure 3, we choose $P_{00}$ and $P_{10}$ as the feature points of mobile robot, and (x,y,θ) indicates the pose of mobile robot and the pose (x,y,θ) of the mobile robot is calculated as follows:

(3) $$\{\begin{array}{l} x=\frac{x_{11}+x_{10}}{2}-\frac{x_{01}+x_{00}}{2}\\ y=\frac{y_{11}+y_{10}}{2}-\frac{y_{01}+y_{00}}{2}\\ \theta =\tan^{-1}\frac{y_{11}-y_{10}}{x_{11}-x_{10}}-\tan^{-1}\frac{y_{01}-y_{00}}{x_{01}-x_{00}} \end{array}$$

where $\left(x_{00},y_{00}\right)$ and $\left(x_{10},y_{10}\right)$ are the initial coordinates of $P_{00}$ and $P_{10}$, respectively, $\left(x_{01},y_{01}\right)$ and $\left(x_{11},y_{11}\right)$ are the coordinates of the corresponding feature points $P_{01}$ and $P_{11}$, respectively (see Figure 3).

## 3. Tracking Algorithm for the Moving Target

To track a fast-moving target with small geometric size, the tracking algorithm requires a fast tracking rate and high detection precision. Because of the circular correlation, the KCF algorithm can expand limited training data by implicitly including all shifted samples of given patches [48,49]. Moreover, the computational effort of training and detection is significantly reduced in the Fourier domain. Owing to these advantages, the KCF is especially suitable for micro-target tracking. However, the periodicity of the circular correlation of KCF produces unwanted boundary effects, which severely limit the target-search region at the detection step. To resolve these inherent problems, we propose the K^2^CF algorithm for moving target tracking. The K^2^CF includes a Kalman predictor that efficiently predicts the positions of the detection samples before the KCF detection. The effect of incomplete negative training samples is weakened by a Gaussian model.

### 3.1. Analysis of the KCF Tracking Algorithm

#### 3.1.1. Training and Detection

The KCF tracking algorithm attempts to train a function $f(z)=w^{T}z$ that minimizes the squared error over the samples $x_{i}$ and their regression labels $y_{i}$. The optimal solution of the optimization problem can be expressed as:

(4) $$\min\sum_{i}\left(f(x_{i}\right)-y_{i}{)}^{2}+\lambda \|\;w\;{\|}^{2}$$

where *λ* is a regularization parameter that controls overfitting (the state vector machine uses a similar parameter). Equation (4) is a linear least-squares problem. To map the inputs of a liner problem to a nonlinear feature space φ(x), define the correlation function as: $k\left(x,x^{\prime }\right)=\varphi^{T}\left(x\right)\varphi \left(x^{\prime }\right)$, we apply the kernel trick and express the solution of w as a linear combination of samples: $w=\sum_{i=1}^{n}\alpha_{i}\varphi (x_{i})$ [50,51]. The function $f(z)=w^{T}z$ is then represented as

(5) $$f(z)=w^{T}z=\sum_{i=1}^{n}\alpha_{i}k(z,x_{i})$$

where z and x denote the candidates detection patches and the training samples, respectively. Consequently, Equation (4) is solved as

(6) $$\alpha ={\left(K+\lambda I\right)}^{-1}y$$

where K is a n×n kernel matrix with elements $K_{ij}=k(x_{i},x_{j})$, y is the regression output of all samples, I is the identity matrix. α is a parameter matrix needs to be optimized.

By Theorem 1 in [38], the cyclic matrix K can be transformed into a diagonal matrix by the discrete Fourier transform (DFT) [52,53]. Accordingly, Equation (6) is solved in the Fourier domain as

(7) $$\hat{\alpha }=\frac{\hat{y}}{{\hat{k}}^{XX}+\lambda }$$

The quantities $k^{\mathrm{xx}}$, expressing the kernel correlations of each x with itself, occupy the first row of the kernel matrix K; the hat symbol ^ denotes the DFT of a matrix.

The parameter α of the classifier function f(z) is obtained through the training samples x. Using the learned function f(z), we evaluate the classification scores of all cyclic shifts of a candidate detection sample:

(8) $$f\left(z\right)={\hat{k}}^{xz}⊙\hat{\alpha }$$

where ⊙ is a dot product of the matrix. The quantities $k^{xz}$, expressing the kernel correlations between z and x, occupy the first row of the kernel matrix $K^{z}$. The position of the shifted samples that maximizes the classification score is the target location. 

After locating the target by Equation (8), we obtain a training sample $x_{k}$ around the target position. The parameter $\alpha_{k}$ of the classifier function f(z) at the current time is computed by Equation (7). The algorithm is updated by the following strategy:

(9) $$\left\{ \begin{array}{l} \alpha_{k}^{\prime }\;=\;\beta \alpha_{k}+(1-\beta )\alpha_{k-1}\\ x_{k}\;=\;\beta x_{k}+(1-\beta )x_{k-1} \end{array}\right.$$

where *β* is the learning factor.

#### 3.1.2. Boundary Effect of KCF

The KCF formulation trains the parameter α of the classifier function f(z) in Equation (7) on the training sample x and its shifted samples. At the detection step (8), the detection samples are also shifted by the base candidate sample z.

**Figure 4:**
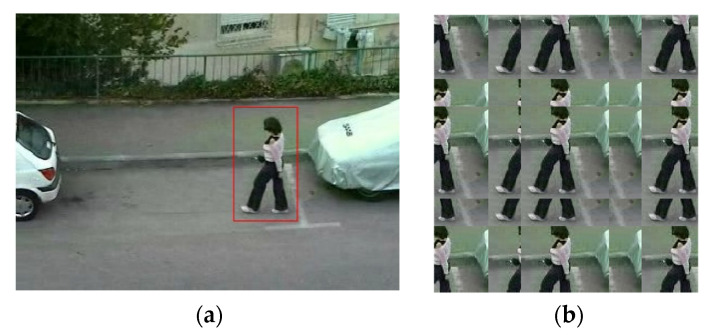
Circular shift: (**a**) original image; (**b**) periodicity in shift samples.

As shown in Figure 4, shifting the base training sample (a) obtains a set of negative training samples (b). However, these negative training samples fail to capture the true image content, because the periodicity of the shift samples introduces a boundary effect that reduces the discriminative power of the learned classifier. To ensure the integrity of the shift samples, we preprocess the target region through a cosine window filter.

**Figure 5:**
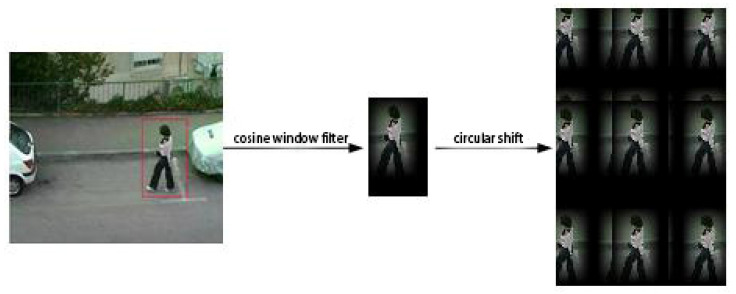
Extraction process of cyclic samples.

**Figure 6:**
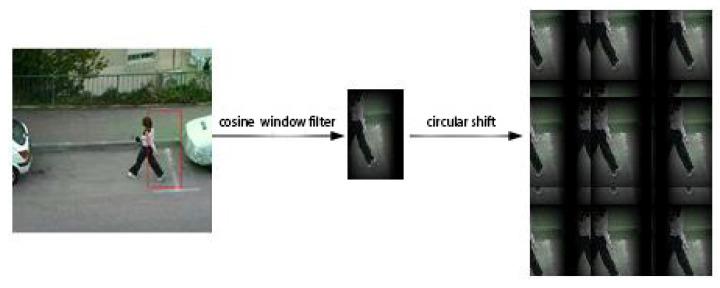
Detection samples of fast target.

Figure 5 is the result of extending the target region and smoothing the edges through the cosine window filter. A circular shift of the base sample then acquires complete negative training samples. However, if the center of the shift target is near the boundary, some incomplete negative samples are also generated. In the detection step, when a fast-moving target is near the boundary of the detection region, the cosine window filter will filter out the target information (see Figure 6).

The above-mentioned limitations of the KCF hamper the tracking performance in several ways. First, KCF-based trackers cannot easily detect fast target motions, as their search regions are limited. Second, any incomplete negative training patches will affect the accuracy of the learned model. Third, the training and detection limitations reduce the re-detection potential of the tracker after interruption by an occlusion.

### 3.2. Framework of K2CF Tracking Algorithm

To resolve the problems caused by the circular shift and cosine window filter, we define the importance of negative training samples by a Gaussian function, and predict the position of the detection target by a Kalman filter [54,55,56].

#### 3.2.1. Training and Prediction

To solve Equation (4) in the KCF, we train the classifier f(z) on a set of training samples ${\left\{\left(x_{i},y_{i}\right)\right\}}_{i=1}^{t}$. Each training sample $x_{i}$ consists of a C-channel HOG feature map. The desired output value $y_{i}$ is the label for each sample $x_{i}$. We define the base sample extracted from the image region as the positive sample and the shift samples as negative samples. To discriminate the incomplete and complete training samples, we calculate the value of the label $y_{i}$, which represents the importance of the training samples, by the following Gaussian function:

(10) $$G(a,b)=\frac{1}{2\pi \sigma_{1}^{2}}e^{\frac{-(a^{2}+b^{2})}{2\sigma_{1}^{2}}}$$

where a and b are the shifted distances of the negative samples along the row and column, respectively. The Gaussian model assigns low importance to shift samples away from the base sample. Incomplete samples, which reduce the discriminant of the classifier model [57,58,59], also receive lower weights. The unequal weights significantly mitigate the emphasis of these shift samples in the learned classifier.

The kernel correlation $k^{xx}$ in Equation (7) is computed with Gaussian kernels $k(x,x^{\prime })=\exp(-\frac{1}{\sigma_{2}^{2}}\|\;x-x^{\prime }\;{\|}^{2})$ as follows:

(11) $$k^{xx^{\prime }}=\exp\left(-\frac{1}{\sigma_{2}^{2}}\left({\left\|x\right\|}^{2}+{\left\|x^{\prime }\right\|}^{2}-2f^{-1}\left(\sum_{C}\hat{x}*⊙{\hat{x}}^{\prime }\right)\right)\right)$$

where $k^{xx^{\prime }}$ expresses the kernel correlation of x with $x^{\prime }$. The Gaussian kernel is less interfered by noise than the linear and polynomial kernels. Using Equations (7), (9), and (10), we can train the parameter α of the classifier function f(z).

The classification scores of all cyclic shifts of test sample z in Equation (8) are evaluated by a learned classifier. The detection base sample z is extracted from the image region that is the target location in the previous frame of the KCF formulation. Therefore, the target can move only within a limited region. 

To solve the above problem, we estimate the position of the test region by a predictor. The target state is denoted by a vector $S=\text{\{}x,y,v_{x},v_{y}\text{\}}$, where x and y are the coordinates of the tracking object center, and $v_{x}$ and $v_{y}$ are the velocity components along the x and y axes, respectively. Following the basic principle of the Kalman filter, the state equation of the discrete dynamic system is recursively obtained as

(12) $$\left\{ \begin{array}{l} s_{k|k-1}=As_{k-1|k-1}+Bu_{k-1}\\ p_{k|k-1}=Ap_{k-1|k-1}A^{T}+Q_{k-1} \end{array}\right.$$

where $s_{k|k-1}$ is the estimated state of the tracking object at time k, $s_{k-1|k-1}$ is the predicted state of the tracking object at time k−1, A and ***B*** are constant matrices describing the state transition and state control of the object, respectively, $u_{k-1}$ is the control vector, $p_{k|k-1}$ is the estimated covariance matrix at time *k*, $p_{k-1|k-1}$ is the predicted covariance at time *k*−1, and $Q_{k-1}$ is the error matrix in the state transition process.

#### 3.2.2. Detection of KCF

To resolve the boundary effect, we predict the location of the detection region by a Kalman filter, which ensures that the candidate detection sample is within the predicted boundary of the region. The candidate test patch z is then extracted at the prediction position $s_{k|k-1}$. Exploiting the cyclic property, a set of detection samples is obtained as

(13) $${\text{\{}P}^{u}z\;|\;u=0,1,\cdots ,n-1\text{\}}$$

where u denotes a shift element. Each sample z is periodically obtained after n shifts.

In Equation (8), the kernel matrix $K^{z}$ denotes the correlation between all training samples X and all candidate patches Z. The samples and patches are cyclic shifts of the base sample x and base patch, respectively. The KCF algorithm processes the kernel matrix $K^{z}$ as a circulant, defined as

(14) $$K^{z}=C(k^{xz})$$

where $k^{xz}$ (occupying the first row of the kernel matrix) denotes the kernel correlation between x and z, and C denotes that $K^{z}$ is generated by shifting $k^{xz}$.

This cyclic matrix property means that $K^{z}$ can be diagonalized by the DFT as

(15) $$K^{z}=Fdiag(\overset{\wedge }{k^{xz}})F^{T}$$

where F is the DFT matrix. Here, the hat symbol ^ and T denote the DFT and conjugate transpose of a matrix, respectively [60,61].

From Equation (5), the correlation values of all detection patches are computed as

(16) $$f(z)={\left(K^{z}\right)}^{T}\alpha$$

where f(z) contains the classification scores of all cyclic shifts of z. Equation (16) can be efficiently calculated by diagonalizing Equation (16) in the DFT domain (Equation (15)). Accordingly, the location of maximum in f(Z) is the target position in the current frame.

#### 3.2.3. Adaptive Optimization of the Position Predictor

Equation (11) gives a preliminary estimate of the state. The target state is then optimized by the real observed value ${ϒ}_{k-1}$, which corrects the estimating error. In the discrete dynamic system, the target state is corrected as

(17) $$\left\{ \begin{array}{l} K_{k}=p_{k|k-1}H^{T}{\left(Hp_{k|k-1}H^{T}+R\right)}^{-1}\\ s_{k|k}=s_{k|k-1}+K_{k}({ϒ}_{k-1}-Hs_{k-1|k-1})\\ p_{k|k}=(I-K_{k}H)p_{k|k-1} \end{array}\right.$$

where $K_{k}$ is the state gain matrix, H is the observation transfer matrix, $p_{k|k}$ is the covariance matrix after correcting the target state, ***I*** is the identity matrix, and R denotes the observation error in the system. Because the imaging system is low-noise, R is set as a constant matrix. Equation (12) corrects the estimated state using the observation error at the previous time. Here, $s_{k|k}$ denotes the predicted position of the detection region in the current frame.

First, the target position is predicted by a Kalman filter. The testing samples around the prediction location are then extracted, and the target in the current frame is located by the KCF tracker. Finally, the predicted target position is refined by the detected value. The authors of [41] selected the position of an occluded target using a threshold value σ. If the detected value of the KCF tracker is less than σ, the Kalman filter is updated using the detected value, and the result is set as the target location. Otherwise, the detected value is ignored and the predicted value of the Kalman filter is set as the target location. However, this tracker is unsuitable for fast-moving targets.

Many fast-motion targets follow nonlinear dynamics. If the target position of a nonlinearly moving object is predicted by a conventional Kalman filter, the predicted state will deviate from the actual state. As the acceleration of the tracked object varies over time, the conversion error matrix $Q_{k-1}$ varies throughout the transition process. The error matrix $Q_{k-1}$ must then be updated by analyzing the target acceleration.

In this system, the mobility of the target is not high, so $Q_{k-1}$ can be updated using the frequency δ and variance $\sigma^{2}$ of the acceleration:

The frequency of acceleration α is computed from three adjacent frames as

(18) $$Q_{k-1}=2\delta \sigma^{2}I$$

(19) $$\delta =\left|\frac{a_{k-1|k-1}+a_{k-3|k-3}-2a_{k-2|k-2}}{T}\right|$$

where $a_{k-1|k-1}$, $a_{k-2|k-2}$, $a_{k-3|k-3}$ indicate the accelerations at three adjacent moments *k*−1, *k*−2, and *k*−3, respectively. The sample interval *T* is usually set to 1.0 [62,63]. When the target is moving at uniform speed, the frequency of the acceleration δ is unchanged, which conforms to actual situations.

The maximum value of f(z) given by Equation (16) is the highest classification score of the cyclic shift samples, which is assigned to the target location in the current frame. In the detection step, the correlation filter detects the candidate patch extracted from the image region at the prediction position. If the predicted position is exactly at the target center, the highest detection score is allocated to the 0-element shift. However, if the predicted position deviates from the target center, the location of the highest detection score is an *n*-element shift. The *n*-element shift denotes the distance between the predicted position and the actual target position. In fact, the predicted and actual target positions usually deviate when the acceleration is variable, indicating that the prediction error is caused by an acceleration disturbance:

(20) $$n=S_{1}(k/k-1)-S_{1}(k)$$

In Equation (20), $S_{1}(k/k-1)$ denotes the predicted position at time *k* from time *k*−1, and $S_{1}(k)$ is the actual detection location at time *k*. Within the sampling period *T*, the error between the predicted and detected positions is related to the acceleration variation Δa as

(21) $$S_{1}(k/k-1)-S_{1}(k)=\frac{T^{2}}{2}\Delta a$$

The acceleration covariance is linearly related to the absolute value of the acceleration variation. In turn, the acceleration variation is linearly related to the position error as follows:

(22) $$\sigma^{2}=\eta \left|\frac{2(S_{1}(k/k-1)-S_{1}(k))}{T^{2}}\right|$$

where *η* is the linear factor. Using Equation (22), we can adaptively adjust the acceleration covariance.

Because the acceleration defines a velocity change, we can update the predictor state and modify the predictor velocity with the prediction error *n*, which is related to the acceleration variation Δa.

(23) $$\left\{ \begin{array}{l} s_{k|k}.v_{x}=s_{k-1|k-1}.v_{x}+n.x\times T\\ s_{k|k}.v_{y}=s_{k-1|k-1}.v_{y}+n.y\times T \end{array}\right.$$

where *n* denotes the shift distance between the predicted and actual positions. Therefore, the actual location of the target in the current frame is determined as

(24) $${ϒ}_{k}=s_{k|k}+n$$

In the K^2^CF, the weights of all shift training samples are determined by the Gaussian model. By assigning lower labels to incomplete samples affected by the boundary, we can reduce the influence of the incomplete samples on the learned classifier model. Prior to detection, the test region is identified by the Kalman filter. At the predicted position, the candidate base patch is extracted from the image region, which ensures that the target lies within the boundary. During the detection step, the location of the maximum f(z) determines the prediction error of the predictor. Knowing the detected position, we can promptly correct the state transfer error and update the target state to optimize the predictor.

## 4. Feature Points for High-Precision Tracking

**Figure 7:**
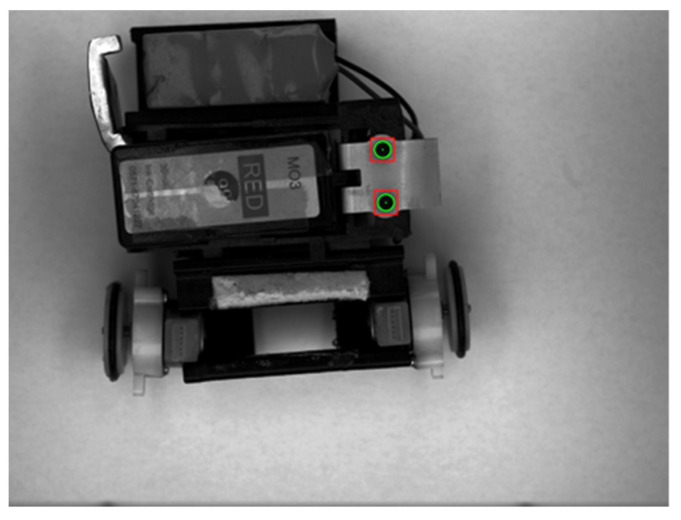
Circular tracking object.

As mentioned in Section 1, the pose measurement of a micro-robot requires two feature points. However, when the target moves outside the boundary, the extracted target is incomplete. Therefore, rather than taking the center of the tracking object, we select the center of the extracted circular features as the feature points on the micro-robot (see Figure 7).

### 4.1. Contour Extraction of a Tracked Object

**Figure 8:**
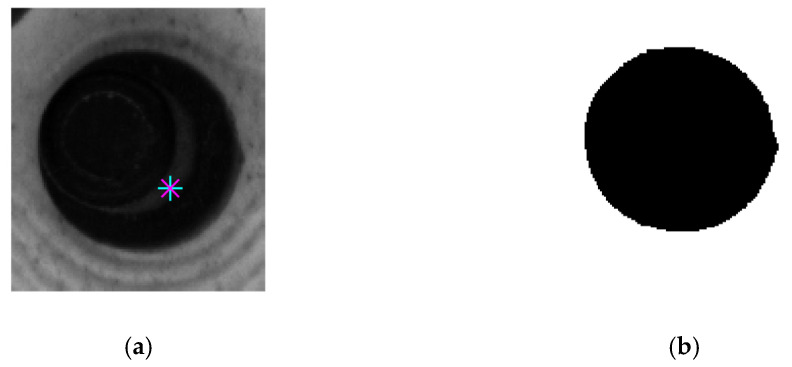
Tracking object segmentation: (**a**) original tracking target; (**b**) segmented circular object.

**Figure 9:**
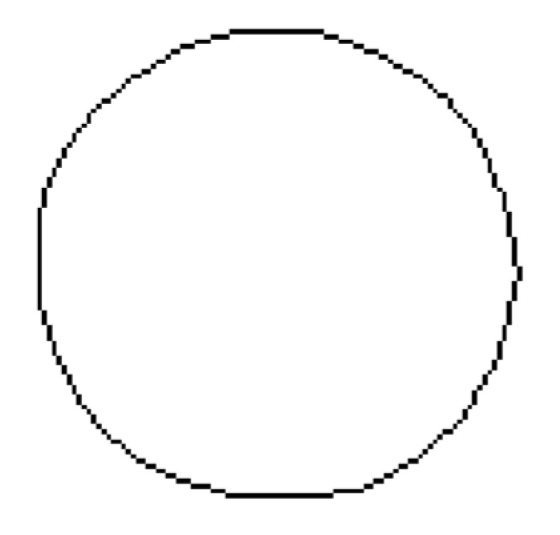
Contour of the tracked circular object in Figure 8.

To extract the contour of a tracked circular object, we first separate the circular object by the region-growth method. As shown in Figure 8a, one point in the circular object is randomly selected as a seed. The region around the seed grows by judging the similarity of the gray value of the neighborhood points. The growth continues until the circular object is completely segmented. After segmenting the circular object, the contour points are extracted by the Canny operator. The result is shown in Figure 9.

### 4.2. Optimization of the Tracked Object Contour

**Figure 10:**
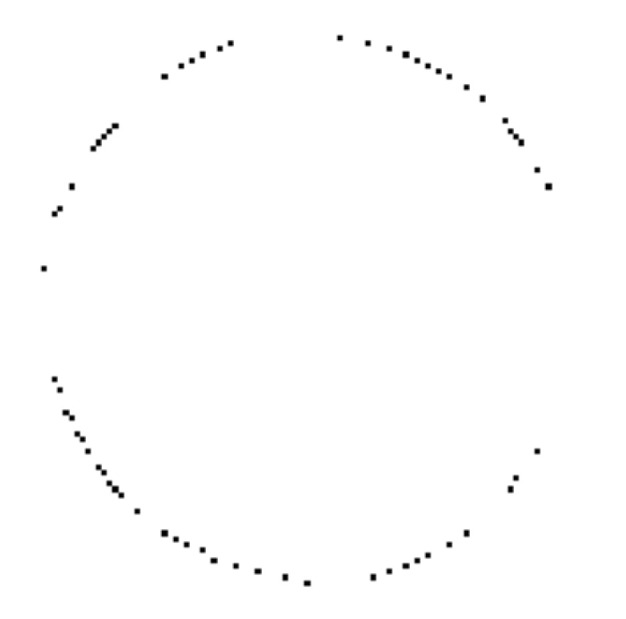
Optimized contour points.

The segmented circular contour is roughened by “burr points” among the extracted contour points. Removing these points will improve the accuracy of the circle fitting. The curvature at the midpoint of three adjacent contour points $Q_{1}(x_{1},y_{1})$, $Q_{2}(x_{2},y_{2})$, $Q_{3}(x_{3},y_{3})$ on the contour is calculated as

(25) $$k=\frac{1}{r}=\frac{1}{\sqrt{{\left(x_{0}-x_{2}\right)}^{2}+{\left(y_{0}-y_{2}\right)}^{2}}}.$$

where ( $x_{0},y_{0}$) is the center of the circle determined by the three adjacent points. When the curvature at a contour point exceeds some given threshold, that point is considered as a “burr point” and is rejected. The optimized contour points are drawn in Figure 10.

### 4.3. Roundness Fitting to the Optimization Contour Points

The optimized contour points are fitted to a circle by the least-squares method. The fitting-circle equation is given by

(26) $${\left(x-x_{c}\right)}^{2}+{\left(y-y_{c}\right)}^{2}=R^{2}$$

where $\left(x_{c},y_{c}\right)$ is the center of the fitting circle (the required feature point), and R is the radius of the fitting circle. The least-squares method minimizes the quadratic sum of the distance from the contour points to the circle center. This optimization problem is given by

(27) $$f=\sum_{j=0}^{n}{\left(\sqrt{{\left(x_{j}-x_{c}\right)}^{2}+{\left(y_{j}-y_{c}\right)}^{2}}-R\right)}^{2}$$

To acquire the extremum of (27), we set the partial derivatives equal to 0.

(28) $$\frac{\partial f}{\partial x_{c}}=0\;,\;\frac{\partial f}{\partial y_{c}}=0$$

**Figure 11:**
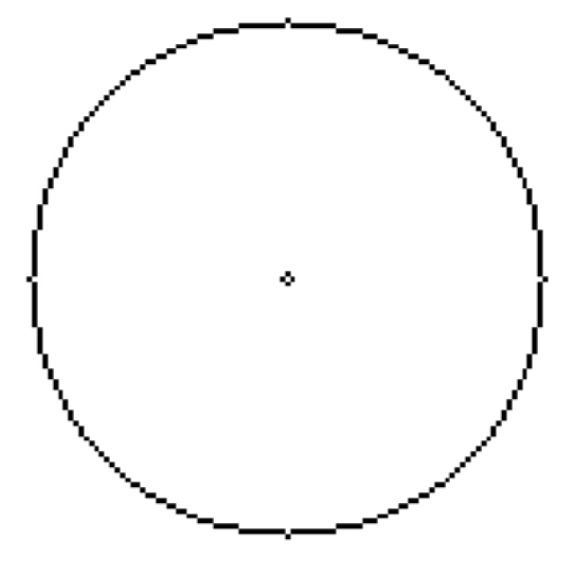
Optimized fitting circle.

The fitting circle obtained by Equation (28), and its center, are shown in Figure 11.

The high-precision feature points are extracted by the region-growth method and the Canny operator as the contour points of the tracked object. These contour points are then optimized by calculating the contour point curvatures. Finally, the optimized contour points are fitted to a circle by the least-squares method, which extracts the precise center of the circle as a feature point.

## 5. Numerical Results and Discussion

### 5.1. Implementation Details

The proposed K^2^CF algorithm was competed against the KCF algorithm and the Kalman filter- based tracking algorithms in several numerical instances. The present trackers were implemented using OpenCV library. In the Kalman filter, the initial motion state was set as s={0,0,0,0} and the transition matrix A was set as

(29) $$A=\left[\begin{array}{cccc} 1 & 0 & \Delta t & 0\\ 0 & 1 & 0 & \Delta t\\ 0 & 0 & 1 & 0\\ 0 & 0 & 0 & 1 \end{array}\right]$$

The conversion error matrix $Q_{k-1}$ follows a metabolic transition process. Its initial value was set as

(30) $$Q_{k-1}=\left[\begin{array}{cccc} 1 & 0 & 0 & 0\\ 0 & 1 & 0 & 0\\ 0 & 0 & 1 & 0\\ 0 & 0 & 0 & 1 \end{array}\right]$$

The observation transfer matrix H was set as a constant matrix.

The performance of our tracker was compared with those of the KCF and Kalman filter-based tracking. All trackers were implemented in the OpenCV library and C++ on Windows 10 running on a computer with an i7 CPU and 16 GB RAM.

To compare the performance of each method in micro target estimation, we constructed numerical motion models under different circumstances in 3D-Max software. These models obtained the actual target position in each frame by detecting a mark along the coordinate axis. Next, we varied the motion states and target speeds in the simulation models. To test the adaptive ability of K^2^CF, the initial states of predictor should also be varied. For a fair comparison, all trackers were given the same sample size and frame sequence. For an intuitive demonstration of the tracking effect, the tracking error was denoted as $△\varepsilon =\sqrt{(x^{\prime }-x{)}^{2}+(y^{\prime }-y{)}^{2}}$, where (x,y) and $\left(x^{\prime },y^{\prime }\right)$ are the true and detected coordinates, respectively.

### 5.2. Tracking Experiment of a Uniformly Moving Target

**Figure 12:**
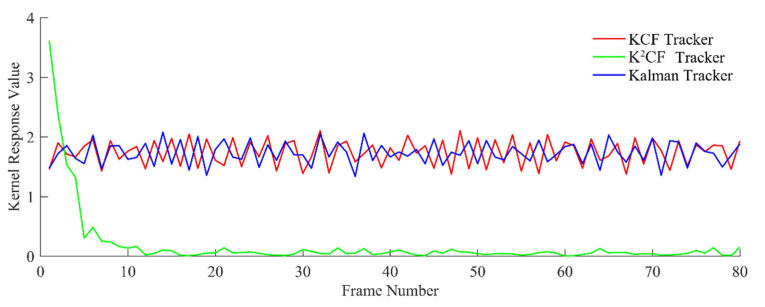
Output detection value of uniform state.

**Figure 13:**
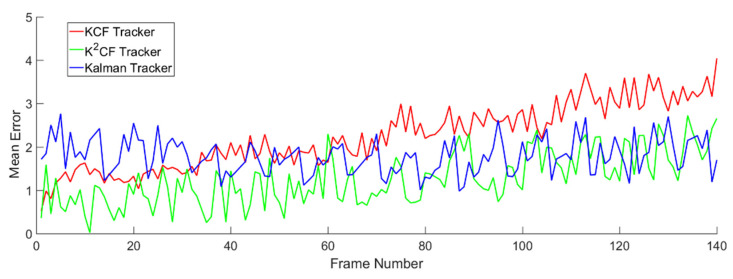
Detection errors of the trackers for a target with uniform velocity.

In the uniform-speed models, we varied the amplitude of the speed in each frame sequence. Because the sampling time between two frames was equal, the moving speed of the target was represented by the displacement of the target between two frames. The detection results are shown in Figure 12 and Figure 13.

Figure 12 shows the output value of the kernel correlation detection. The values were almost unchanged across the frame sequence, confirming a constant displacement between pairs of frames. These results reflect the actual motion state. The output value of K^2^CF rapidly converged to 0, confirming that the Kalman predictor in K^2^CF adjusted the initial prediction speed to the actual speed using the kernel correlation filter. In contrast, the output value of the Kalman tracker converged to a nonzero value, indicating that the Kalman predictor alone cannot adjust the prediction state. Meanwhile, the KCF tracker has a fixed detection range, so its kernel response value was almost constant at some value above 0.

Figure 13 shows the detection error of the three trackers. The mean errors in the KCF, Kalman and K^2^CF trackers were ${\bar{\Delta }}_{1}=0.9597$ pixels, ${\bar{\Delta }}_{2}=0.9290$ pixels, and ${\bar{\Delta }}_{3}=0.9074$ pixels, respectively. Apparently, K^2^CF achieved a higher detection precision than the other trackers.

Next, the tracking effects of the three trackers were tested on a fast-moving target. For this purpose, a target of radius 3 mm was moved at speeds of $\left\{v_{1},2v_{1},3v_{1},4v_{1},5v_{1},6v_{1},7v_{1}\cdots \right\}$, where $v_{1}=\sqrt{v_{x}^{2}+v_{y}^{2}}$, $v_{x}=1\;\mathrm{mm}/s,\;v_{y}=0.5\;\mathrm{mm}/s$. The average detection errors of the KCF, Kalman, and K^2^CF trackers were obtained and reported at each speed.

**Figure 14:**
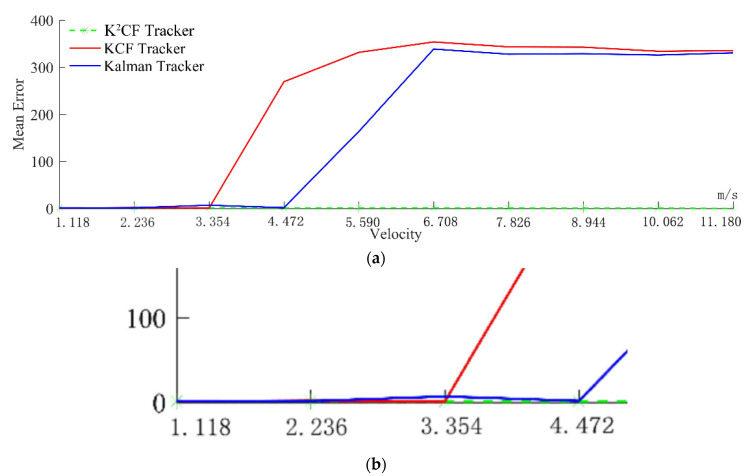
Detectable velocity of uniform state: (**a**) the mean error of velocity from 1.118 m/s to 11.180 m/s; (**b**) amplification of velocity error from 1.118 m/s to 4.472 m/s.

When the velocity exceeded 3v, the tracking error of KCF increased sharply and the tracking failed (Figure 14a). The graduations of the velocity axis is clearly seen in Figure 14b. The Kalman tracker exhibited similar behavior. In contrast, the average error of the K^2^CF tracker was less than 2 pixels at any target speed, and even the fastest-moving object was stably tracked. This result confirms that the K^2^CF tracker perfectly combined the Kalman predictor with the kernel correlation detector, and hence diminished the boundary effect during fast tracking.

### 5.3. Tracking Experiment with a Uniformly Accelerating Target

**Figure 15:**
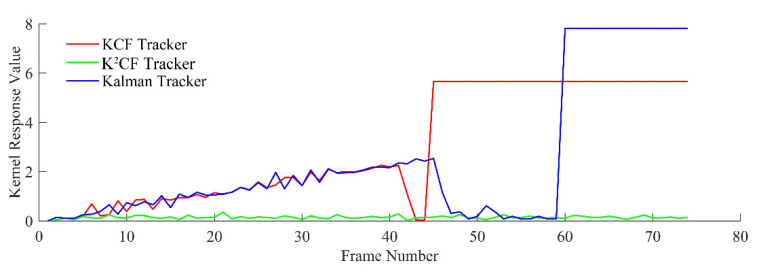
Output detection value of uniform acceleration state.

**Figure 16:**
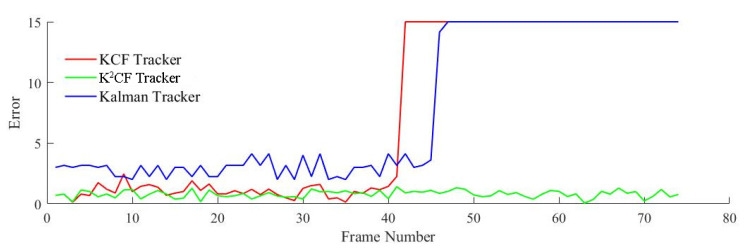
Detection error of uniform acceleration state.

In the uniform acceleration model, the accelerated speed was set to $a_{x}=0.21,a_{y}=-0.1$, the initial velocity was $v_{x}=0.1,v_{y}=-0.4$, and the target radius was 3 mm. The detection results of the three filters are shown in Figure 15 and Figure 16.

The moving distance of the target between two frames increased with velocity. As shown in Figure 15, the output values of the KCF and Kalman trackers initially increased, but (as explained in Section 2), the boundary effect of the KCF causes target loss when the target moves outside the detection range. After frame 42, the KCF tracker lost the target because the object was moving too quickly. The Kalman tracker also lost the target, because its predicted speed cannot adaptively adjust. However, the output value of the K^2^CF tracker was always close to 0, demonstrating that the Kalman filter effectively resolved the boundary effect of kernel correlation detection.

The detection errors of the KCF and Kalman trackers increased steeply at frame 42, and the target was lost shortly afterward. However, the K^2^CF tracker maintained continuous tracking with an average error of 0.7956.

To verify the adaptive effect of the K^2^CF tracker, we then changed the initial state of the predictor. By observing the predictor speed throughout the tracking process, we can certify the correction effect.

**Figure 17:**
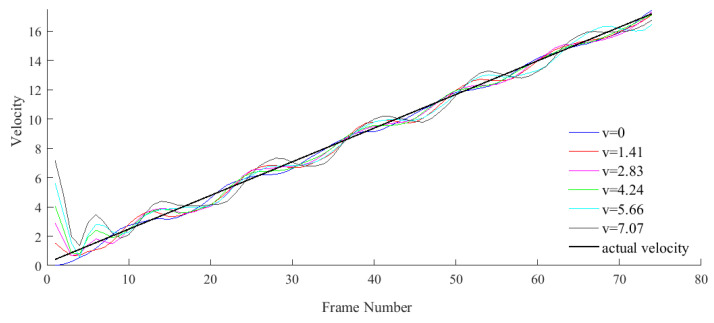
Adaptive velocity adjustment.

In Figure 17, for different initial velocities, the Kalman predictor in the K^2^CF tracker can automatically correct the error between the setting velocity and actual velocity. Nevertheless, the correction range is related to the detection range of the kernel correlation filter according to Equation (23). If the initial error exceeds the target sample size, the Kalman predictor will lose efficacy.

As shown in Figure 17, the Kalman predictor in the K^2^CF tracker automatically corrected the error between the set and actual velocities, regardless of the velocity magnitude. Nevertheless, the correction range was related to the detection range of the kernel correlation filter, as dictated by Equation (23). If the initial error exceeds the correction range, the Kalman predictor will lose its efficacy.

### 5.4. Tracking Experiment of a Nonuniformly Accelerating Target

**Figure 18:**
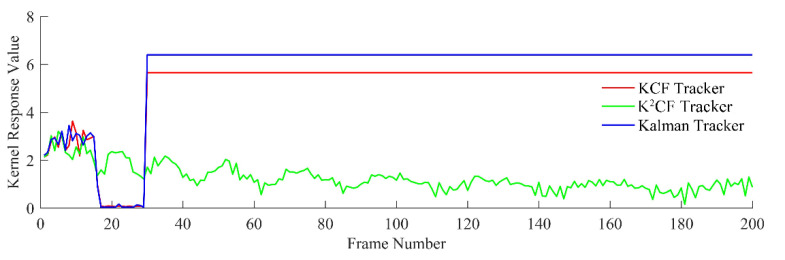
Kernel response value of nonuniform state.

Actual objects do not move with an ideal uniform speed or acceleration because they are interfered with by system noise. Actual accelerations are nonuniform and affected by different disturbances. Therefore, the tracking performances of KCF and K^2^CF were tested in a helix motion model with constantly changing accelerations and velocities. (See Figure 18).

**Figure 19:**
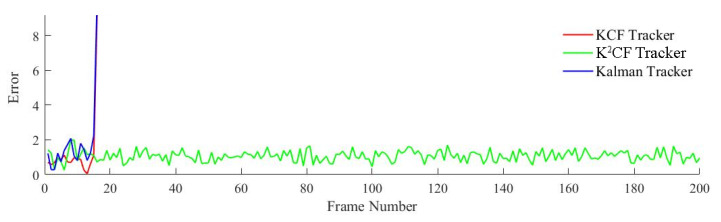
Detection error of nonuniform acceleration state.

Changing the target speed changed the displacement of the target between two consecutive frames. The output values of these trackers varied accordingly (Figure 18 and Figure 19). When the target moved too quickly, it escaped the detection boundary of the kernel correlation filter, and the KCF tracking failed. Meanwhile, the Kalman tracker could not correct the predictive state in response to the motion variations. Therefore, it easily lost the nonlinearly moving target. Conversely, the Kalman predictor in the K^2^CF tracker constantly corrected the predicted moving state, and the kernel correlation detection correctly predicted the target position. Therefore, the target remained within the boundary of the detection region and was continuously tracked. The mean tracking accuracy of the K^2^CF tracker was 1.0704 pixels.

### 5.5. Baseline Comparison

**Figure 20:**
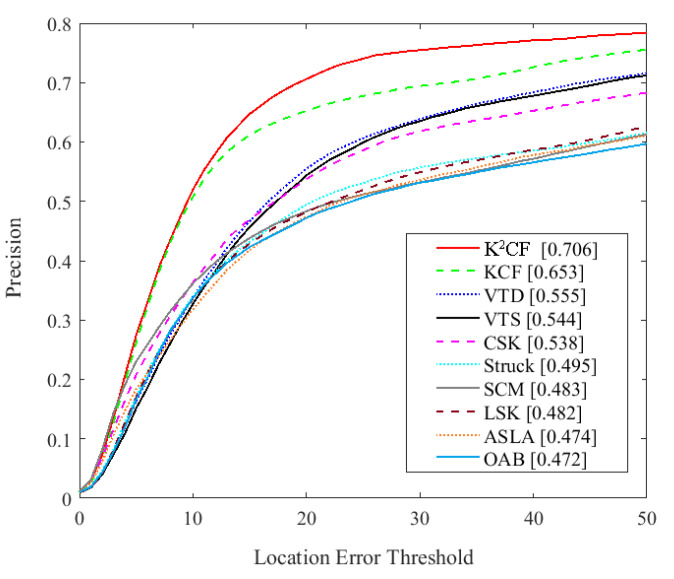
Comparison of state-of-the-art trackers.

The performances of K^2^CF and various state-of-the-art methods were compared on OTB-50 and OTB-100 video sequences extracted from the OTB-2013 dataset. These videos present different tracking challenges, such as illumination variation, rotation, scale change, motion blurring, occlusion and fast motion. Figure 20 shows the location precision curves of K^2^CF and the existing trackers: KCF, visual tracking decomposition (VTD) [64], visual tracking by sampling (VTS) [65], cyclic kernel tracking detection (CSK) [36], structured output tracking with kernels (Struck) [66], the sparsity-based collaborative model (SCM) [67], local sparse and K-selection tracking (LSK) [68], adaptive structural local sparse appearance (ASLA) [69] and online Ada boosting (OAB) [70].

As shown in Figure 20, the K^2^CF achieved the highest mean precision score (0.706) among the tested methods, followed by the KCF. This result confirms the high detection precision of the kernelized correlation filter. Therefore, the KCF-based method can precisely track the moving target.

**Figure 21:**
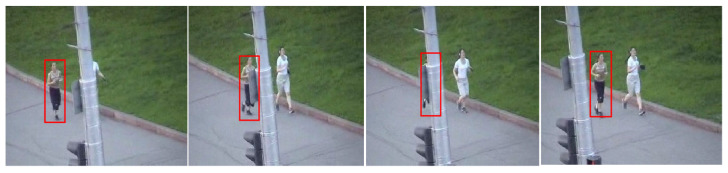
Tracking object for occlusion case.

**Figure 22:**
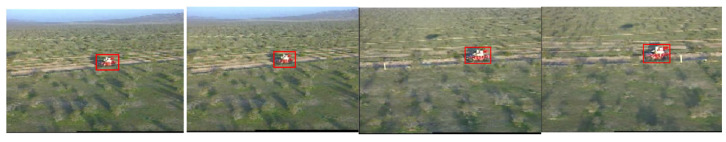
Tracking object for scale change case.

Because the KCF tracker is sensitive to fast motion, occlusion and scale change, the performance of K^2^CF was tested in these scenarios. As the test sample, we extracted the “Jogging” and “RedTeam” sequences from the OTB-100 sequences. Our tracker coped well with both occlusion (Figure 21) and scale changes (Figure 22).

As the K^2^CF tracker delivers its best performance on fast-moving smaller objects, further analysis is required. Using the visual platform designed in Section 2, we collected pictures of the moving micro-robot at a sampling frame rate of 1.0 frame/s.

**Figure 23:**
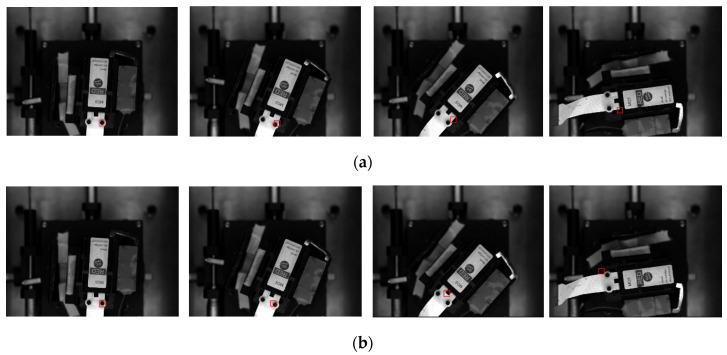

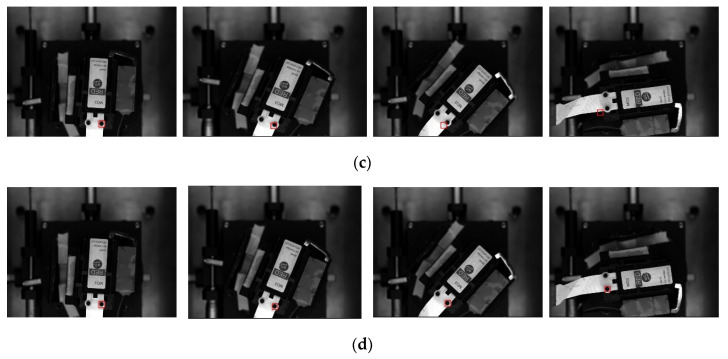
Comparing the trackers’ performance for micro-object fast motion case: (**a**) MOSSE tracker starts to fail at these frames; (**b**) improved KCF tracker starts to fail at these frames; (**c**) KCF tracker starts to fail at these frames; (**d**) K2CF tracker is successful at these frames.

The KCF tracker, the minimum output sum of squared error (MOSSE) tracker, and the improved KCF tracker proposed in paper [41] could not track the micro-object correctly, whereas our tracker successfully tracked all frames (Figure 23). The established trackers coped poorly with the fast motions of the micro-object.

Our tracker markedly outperformed the KCF, MOSSE and the existing improved KCF tracker, particularly when tracking fast-moving micro-objects. We then positioned the micro-robot with the *X–Y–θ* triaxial mobile platform and obtained the ground truth location of the small object. The tracking speeds (average frame rates) and mean precisions of the four trackers are listed in Table 1.

As shown in Table 1, K^2^CF achieved the highest precision and a mean speed of 110.67 fps. Although KCF operated at 125.64 fps, our tracker is suitable for real-time applications while also improving location precision.

## 6. High-Precision Pose Measurement Experiments

### 6.1. Calibration of the Experimental Platform

**Figure 24:**
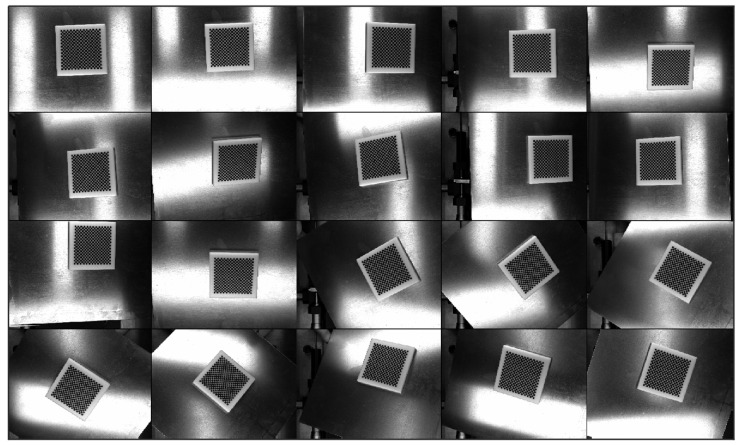
Calibration pattern of camera system.

The effectiveness of the proposed K^2^CF algorithm was checked on an experimental calibration platform. Following the observation method in [71], we employed a (63 × 63 × 6) mm^3^ ceramic checkerboard with a precision of 2 μm as the calibration plate. The calibration plate was photographed from different viewpoints relative to the camera (Figure 24).

From the calibration operation, we obtained the inner parameter matrix $M_{1}$, the outer parametric rotation matrix $R_{1}$, and the translation vector ***T***:

(31) $$\left\{ \begin{array}{l} M_{1}=\;[\begin{array}{ccc} 10313.8 & 0 & 2006.7\\ 0 & 10313.9 & 1565.8\\ 0 & 0 & 1 \end{array}]\\ R_{1}=[\begin{array}{ccc} 0.0003 & 0.9997 & -0.0217\\ 0.9975 & -0.0018 & -0.0694\\ -0.0694 & -0.0217 & -0.9974 \end{array}]\\ T=[-24.3039\;\;-18.7007\;\;467.3700\;] \end{array}\right.$$

**Figure 25:**
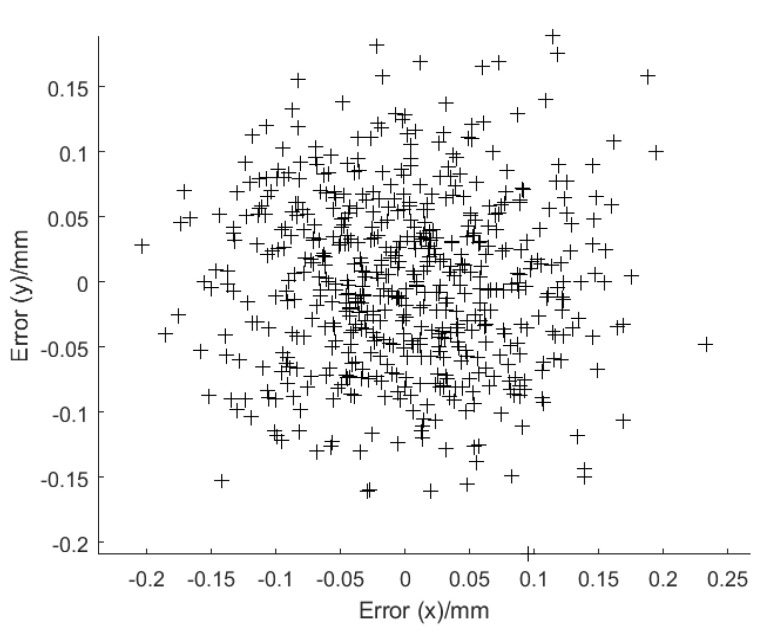
Error distribution of coordinate transformations.

Using the rotation matrix ***R*** and the translation vector ***T***, we then established the mapping relationship between the image and motion coordinate planes. Because the actual coordinates of the chessboard were known, we extracted the image coordinates of the corner points and calculated them in the moving plane. Comparing the calculated and actual coordinates, we finally obtained the coordinate conversion error. From Figure 25, the average errors in the coordinate transformation were determined as error_x = 0.044 mm and error_y = 0.037 mm.

### 6.2. Pose Measurement Precision Experiment

To test the precision of the pose measurement system, we should also obtain the ground-truth location of the micro-robot. The *X–Y–θ* triaxial mobile platform locates the micro-robot with a position precision of 10 μm and a rotation precision of 0.1°. Therefore, we can monitor the ground truth pose of the micro-robot as it moves. Comparing the ground-truth pose with the measured pose, we can obtain the precision of the pose measurements.

Because the Kalman predictor corrects the predicted motion error in any state, the kernel correlation filter can adapt to different moving states of the target. The correction ability of the Kalman predictor is related to the range of kernel correlation detection. Acceleration interference increases the displacement between the frames, so the tracking target will be lost when the increment of the acceleration displacement exceeds the detection boundary.

In the pose-measurement experiment, we varied the inter-frame acceleration displacement s between the images. Here, $s=\sqrt{x^{2}+y^{2}}$, where $x=x_{i}-x_{i-1},y=y_{i}-y_{i-1}$ are the acceleration displacements in the x and y directions, respectively. The target was tracked by the K^2^CF algorithm, and the error e of the system pose measurement was calculated as $e=\sqrt{{\left(△x\right)}^{2}+{\left(△y\right)}^{2}}$, where △x and △y are the errors in the x and y directions, respectively. The acceleration displacement between the frames was then calculated.

**Figure 26:**
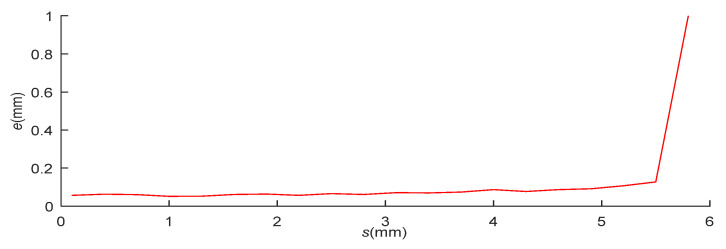
Detection error versus acceleration displacement of the pose measurement system.

As shown in Figure 26, the target was lost when the acceleration displacement between two frames exceeded 5.6 mm. However, at such large acceleration displacements, the maximum inter-frame displacement of the target can reach 8 mm. The pose measurement platform satisfies the moving speed requirements of the micro-robot.

**Figure 27:**
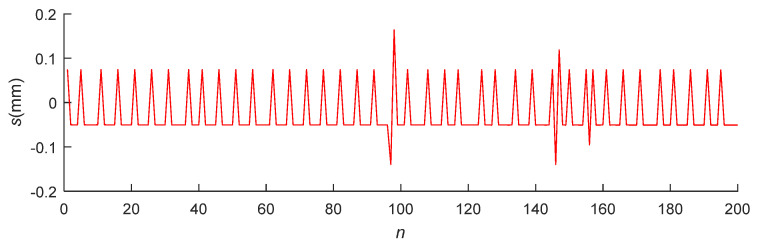
Position detection error of the pose measurement system.

**Figure 28:**
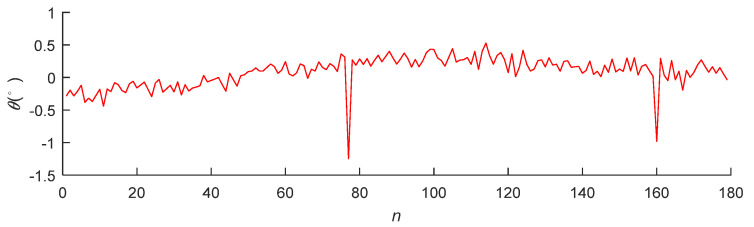
Angle detection error of the pose measurement system.

Figure 27 and Figure 28 present the position error *s* and attitude error *θ* in the actual pose measurements, respectively. The average accuracies of the position and angle measurements were 0.0729 and 0.0824°, respectively, satisfying the precision requirements of the system.

## 7. Conclusions

To mitigate the error fluctuations and tracking losses in high-precision pose measurements of a moving target, we proposed the K^2^CF algorithm. By removing the boundary effect of the KCF algorithm, K^2^CF accurately tracks a fast-moving micro-target. The pose of a micro-robot in a two-dimensional plane was monitored on a visual test platform. Referring to the research goals, the main conclusions are summarized below.

1. A mapping relationship between the moving and image coordinate systems was constructed using the camera calibration principle. A kernel relation filter and a Kalman filter were combined into an adaptive tracking model. The object features were extracted by the region growth method.
2. The feature profile was extracted by the Canny operator, and the feature points were precisely extracted by optimizing the edge points using the least-squares method.
3. The boundary effect of the KCF algorithm was resolved by a Kalman filter that predicts the target position and ensures that the candidate samples lie within the boundary. Comparative simulations confirmed that the proposed K^2^CF algorithm adapts to different moving states of the target and corrects the initial velocity error.
4. The precision of the system was experimentally evaluated on a small target of radius 3 mm. For acceleration displacements lower than 5.6 mm, the average position and angle accuracies were 0.0729 mm and 0.0824°, respectively.

In summary, this paper provides an adaptive method for tracking a moving target. The pose detection system lays a foundation for the trajectory planning of micro-mobile robots. The fast target tracking algorithm achieves precise and stable tracking with a certain versatility. However, stable and accurate tracking is difficult to achieve in the long term because it is interfered with by target occlusion and scale change. Solving these problems to maintain long-term stability and high-precision tracking is our future research goal.

## Figures and Tables

**Table 1:** Average precision and frame fps of 4 trackers.

	Mean of Precision (%)	Standard Deviation of Precision (%)	Mean of fps	Standard Deviation of fps
**K^2^CF tracker**	96.45	6.7	118	110.67
**Improved KCF**	78.35	17.5	125	111.76
**KCF**	80.28	33.67	135	125.64
**MOSSE**	65.17	36.89	254	207.4
